# Chronic cranial window for photoacoustic imaging: a mini review

**DOI:** 10.1186/s42492-021-00081-1

**Published:** 2021-05-26

**Authors:** Yongchao Wang, Lei Xi

**Affiliations:** 1 School of Physics, University of Electronic Science and Technology of China, Chengdu, 610054 Sichuan China; 2 Department of Biomedical Engineering, Southern University of Science and Technology, Shenzhen, 518055 Guangdong China

**Keywords:** Photoacoustic imaging, Photoacoustic microscopy, Long term, Chronic, Cranial window

## Abstract

Photoacoustic (PA) microscopy is being increasingly used to visualize the microcirculation of the brain cortex at the micron level in living rodents. By combining it with long-term cranial window techniques, vasculature can be monitored over a period of days extending to months through a field of view. To fulfill the requirements of long-term in vivo PA imaging, the cranial window must involve a simple and rapid surgical procedure, biological compatibility, and sufficient optical-acoustic transparency, which are major challenges. Recently, several cranial window techniques have been reported for longitudinal PA imaging. Here, the development of chronic cranial windows for PA imaging is reviewed and its technical details are discussed, including window installation, imaging quality, and longitudinal stability.

## Introduction

### Cranial windows for longitudinal brain imaging

**Fig. 1:**
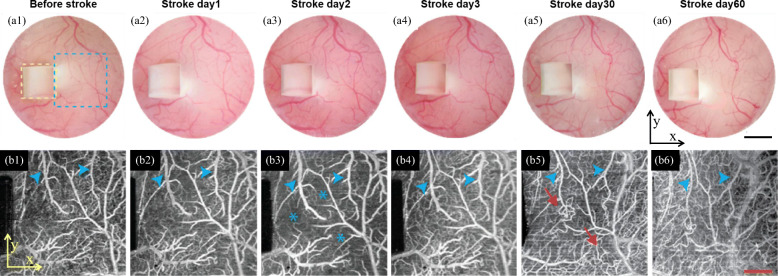
Longitudinal monitoring of acute (day 1 to 3) and chronic (day 30 and 60) changes after transient middle cerebral artery occlusion (tMCAO) [43]. a1-a6 Six optical microscopic images of the cranial window before and after tMCAO. The yellow dashed square represents a microprism. b1-b6 Six top-view visible optical coherence tomography angiography en face images of the region marked by blue dashed square in a1. Black scale bar: 1 mm; red scale bar: 400 μm

As observed in practical cerebrovascular studies, the blood vessels are typically covered with a layer of skull bone, ranging in thickness from several hundred microns to a few millimeters, which is an enormous constraint in brain investigations [1–3]. In the early nineteenth century, the cranial window technique has been introduced for longitudinal brain imaging [4, 5]. Especially with the development of optical imaging techniques, including but not limited to two-photon microscopy [6–14], laser speckle contrast imaging (LSCI) [15–24], and optical intrinsic imaging [25–33], an increasing number of cranial window techniques have been introduced and integrated with these imaging modalities for visualizing the microcirculation of the brain cortex over an extended period of months [3, 34–43]. For example, Morii et al. [34] established a glass-based imaging window to analyze the reactivity of rat pial vessels to adenosine and carbon dioxide. To visualize calcium in virally transfected striate cortical neurons in behaving monkeys, Heider et al. [44] developed a thin-silicone-based artificial dura for two-photon imaging. In addition, Yedid and Bell [37], and Drew et al. [38] developed and improved the thin-skull technique in mice, allowing an extended period of imaging without causing an inflammatory response. Therefore, the cranial window provides a mature access method for a direct, longitudinal, in vivo study of brain functions (Fig. 1).

### Photoacoustic imaging for cerebrovascular visualization

Photoacoustic imaging (PAI) is a hybrid imaging modality that combines the deep tissue penetration capability of ultrasound imaging and the rich contrast and spectral features of optical imaging [45–53]. Generally, in PAI, the object is illuminated with short pulses or amplitude-modulated light, which thermally expands by absorbing and converting light energy into heat, simultaneously with the generation of acoustic waves. The amplitude of the generated acoustic pressure can be expressed as:

$$ P\left(\overrightarrow{r}\right)=\eta \Gamma {\mu}_aF\left(\overrightarrow{r}\right) $$

Here *η* is the conversion efficiency of the absorbed light energy that is converted into heat, Γ is the Grüneisen parameter, *μ*_*a*_ is the absorption coefficient, and *F* represents the local optical fluence.

**Fig. 2:**
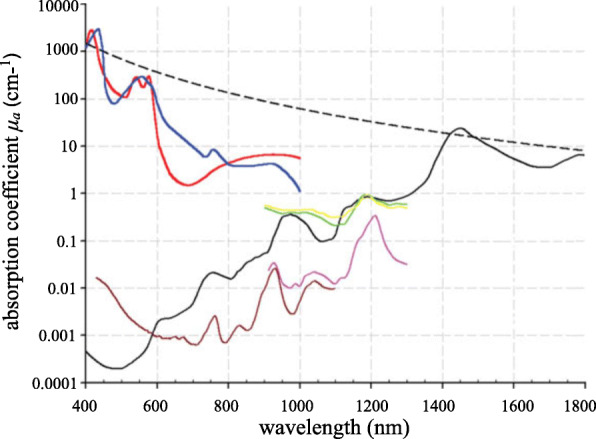
Absorption spectra of endogenous tissue chromophores [54]. Oxyhemoglobin: red line; Deoxyhemoglobin: blue line; Water: black line; Lipid **a**: brown line; Lipid **b**: pink line; Melanin: black dotted line; Collagen: green line; Elastin: yellow line

**Fig. 3:**
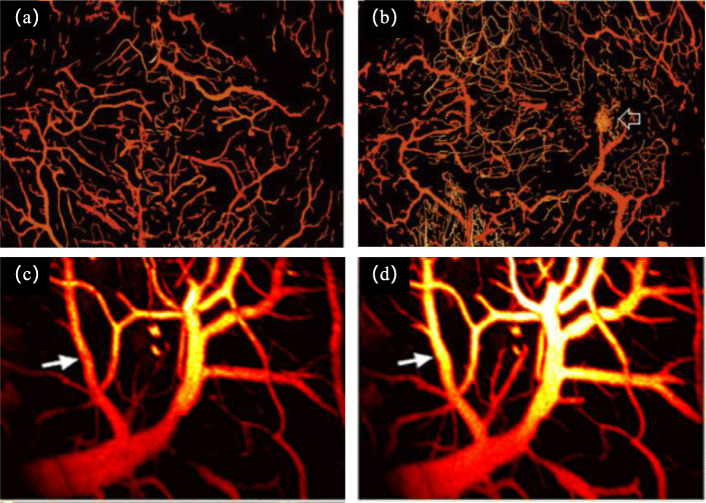
In vivo imaging of cerebrovascular disease model using optical-resolution photoacoustic microscopy (OR-PAM). **a**, **b** PA images of normal and glioma mouse brains, respectively [55]. The tumor core is indicated by the arrow in b. **c**, **d** PA images of mouse brain under ischemia and perfusion, respectively [57]. The white arrows reveal an obvious change in diameter and intensity of a single vessel due to ischemia and perfusion

The amplitude of acoustic pressure is proportional to the absorption coefficient, which is the source of the PAI contrast mechanism. Figure 2 shows the absorption coefficient spectra of various endogenous tissue chromophores [54]. Within the wavelength range of 400 to 600 nm, PAI is extremely sensitive to both oxyhemoglobin and deoxyhemoglobin, proving its superior capability in vascular imaging. In the past decades, PAI has been utilized by numerous groups and has found comprehensive application in cerebrovascular imaging, including brain tumor model [55], epilepsy [56], ischemia-perfusion [57], Alzheimer’s disease [58], stroke [59], and brain edema [60], enabling a better understanding of brain activities and functions (Fig. 3).

## Cranial windows for long-term PAI

With the development and maturity of PA techniques, there is an urgent need for long-term PAI. Hence, investigations using the PA cranial window have attracted increasing attention in the previous decade. However, to date, only a few reports on the cranial window for long-term PAI, which is mainly limited by the window material, have been presented. In pure optical imaging modalities, the target is usually illuminated with a light source and the image is reconstructed by detecting the reflected or stimulated light. Therefore, the cranial window only needs to have sufficient optical transparency and biocompatibility. In contrast to the case in optical imaging, a PA image is obtained by detecting the light-induced acoustic signal, which may undergo prominent attenuation in case of acoustic impedance mismatches between the cranial window and brain tissues. Hence, the cranial window for PAI must simultaneously provide sufficient acoustic transparency. Several chemical and surgical methods have been proposed to overcome this challenge in the PA cranial window [42, 61, 62].

## Skull window technique based on chemical penetration

### Optical cleaning of skull window for PAI

The optical cleaning technique has been widely applied to PAI to enhance imaging quality. Based on the principle of optical cleaning, the sample is usually infiltrated with high-refractive-index optical cleaning agents with improved optical scattering and acoustic impendence properties, enabling sufficient transmission of both light and the induced acoustic wave. For instance, Zharov et al. [63, 64] successfully combined this technique with PA flow cytometry to improve the sensitivity and resolution for the detection of circulating tumor cells in deep blood vessels. Zhou et al. [65] demonstrated that a glycerol–water solution, a commonly used optical clearing agent in tissue optical clearing (TOC), could improve the imaging depth and lateral resolution of OR-PAM. However, all these studies focused on the optical cleaning of skin tissues and were not applied to the skull window for brain imaging.

**Fig. 4:**
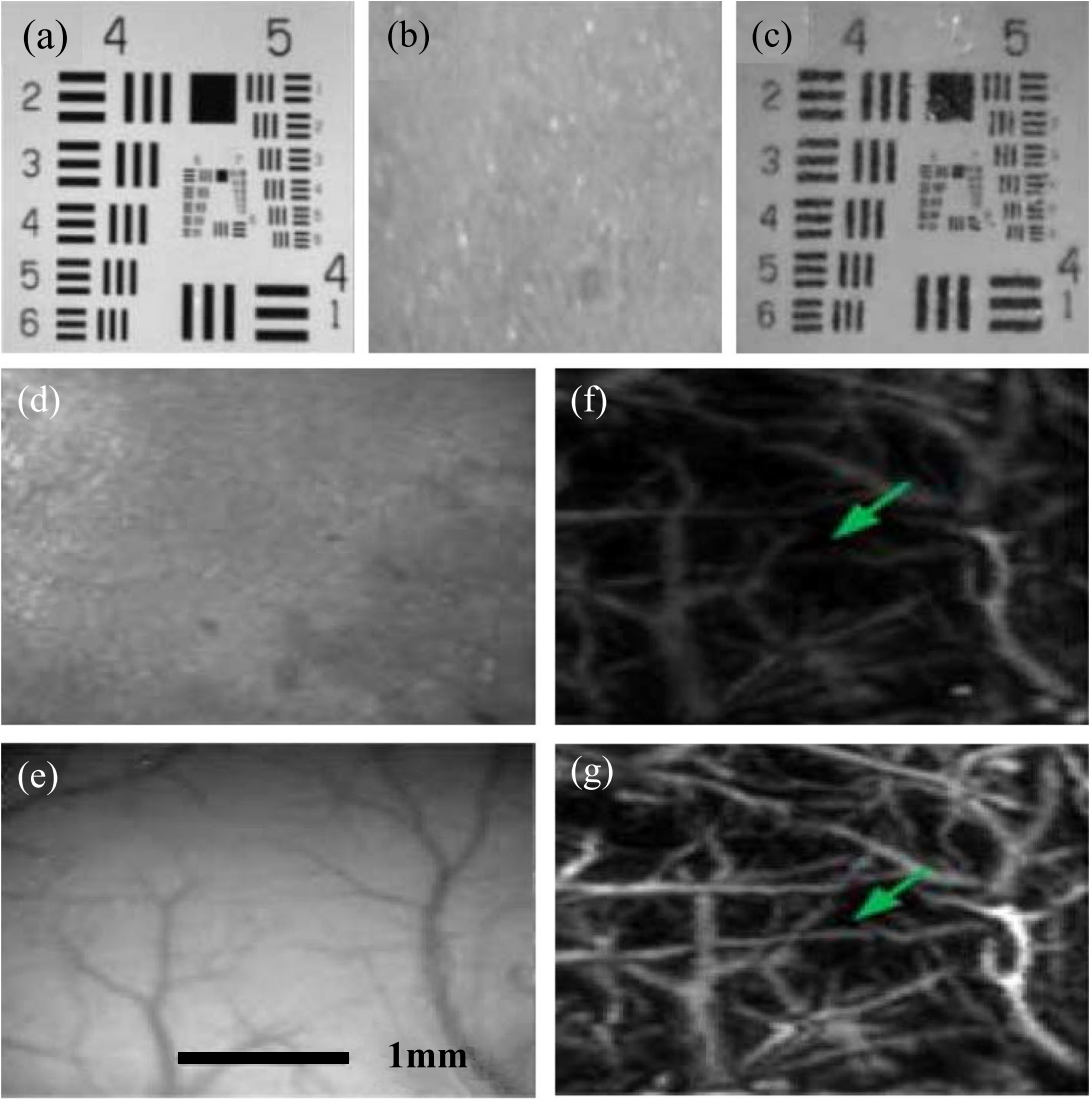
The effect of SOCS on the skull in vitro and in vivo [61]. **a**-**c** Typical reflected images of the USAF target without and with the skull, and with SOCS-treated skull, respectively. Typical reflection images of cortical blood vessels obtained from untreated skull **d** and from the transparent skull after SOCS treatment for 25 mins **e**. Photoacoustic images obtained from intact skull **f** and from the transparent skull after SOCS treatment for 25 mins **g**

In 2008, Genina et al. [66] first applied propylene glycol to the cranial bone in vitro, and the results revealed that both the absorption and scattering coefficients of the sample were reduced by 20%–30%. In the following decade, the optical clearing technique for the skull was further improved and applied to in vivo long-term optical imaging. In 2012, Wang et al. [3] first applied an innovative skull optical clearing solution (SOCS) to living animals. The skull can rapidly become completely transparent with SOCS treatment, enabling high-resolution LSCI of cortical cerebrovascular region. In 2015, Zhang et al. [67] conducted a quantitative analysis of the optical cleaning efficiency of SOCS, demonstrating that the scatter coefficient of the skull was reduced three-fold after SOCS treatment. Based on the innovative SOCS, they further improved the concentration of several components, realizing a rapid and repeatable skull [68]. The results of both white-light and LSCI images revealed that the transparency of the skull was maintainable for 1 h. Post treatment with physiological saline, the skull returned to its initial state. In addition, when the skull is retreated with SOCS, it gains transparency within 10 min, enabling repeated imaging of the cortex vasculature. In 2016, Yang et al. [61] first applied SOCS to enhance the PA image quality of cerebrovascular regions in living mice with intact skulls. They demonstrated that this agent can enhance the transmission of both light and acoustic waves in the skull in vitro. Figures 4(a-c) depict the typical reflected images of the United States Air Force (USAF) target. The target region, initially visible to the eyes, becomes completely invisible when covered with an intact skull. Post treatment with SOCS for 25 mins, the skull becomes optically transparent, enabling clear visualization of the target. The quantitative analysis of the optical transmittance with the skull shows that the transmission efficiency is almost tripled after the application of SOCS. In addition, to evaluate the impact of the SOCS on the ultrasonic transmittance, Yang et al. also performed in vitro ultrasonic experiments by scanning a metal plate beneath the intact skull pre- and post-treatment. The quantitative analysis of the echo signal indicated that the ultrasonic transmittance can be increased 1.58 folds post treatment.

Furthermore, to evaluate the skull cleaning performance in living animals, the pre- and post-treatment imaging quality of the cerebral vasculature in mice with intact skull were compared in detail. As shown in Figs. 4(d-g), both the reflection and PA images revealed that the number of captured vessels increase significantly after SOCS treatment. The PA signal of selected blood vessels was also analyzed, which indicated that their average amplitude increased 2.59 folds, even up to 3.20 folds for a few vessels in the deep tissue. Therefore, it can be concluded that optical cleaning of the skull window can significantly enhance the acoustic-resolution photoacoustic microscopy performance in mice with intact skulls. Repeated treating of the skull enables long-term PA brain imaging in mice with enhanced quality.

## Craniotomy-based chronic cranial window

### Ultrasound-sensing chronic cranial window with soft-nanoimprinting

**Fig. 5:**
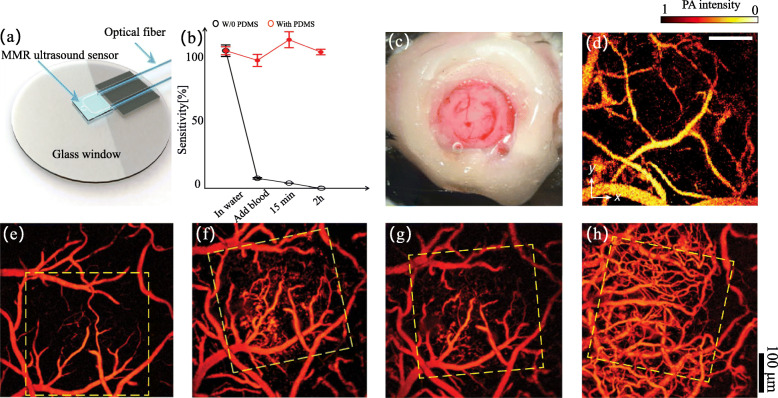
**a** A schematic diagram of the ultrasound-sensing cranial window implant [42]. **b** Comparison of MRR Q-factors with and without the protection layer in whole blood. **c** A photograph of a fabricated chronic cranial window in mice. **d** A typical PA image of cortical vasculature in mice. **e**-**h** PA images of the cortical vasculature in the same area over a period of 28 days

In the previous decade, optically transparent micro-ring resonator (MRR)-based ultrasonic detectors have been developed and integrated into PAI [69–71]. Although the MRR offers promising potential for chronic cranial window owing to its distinct optical transparency and miniaturized form factors, its application in in vivo studies remains unreliable because of the easy contamination of features, poor sensitivity, high cost, and low yield [69, 70]. In 2019, Li et al. [42] introduced an integrated transparent nanophotonic ultrasonic detector and successfully applied it to an ultrasound-sensing chronic cranial window. As shown in Fig. 5a, the ultrasonic detector comprises a glass window, MRR ultrasound detector, and optical fiber.

By fabricating an MRR ultrasonic detector with low-cost soft nanoimprint lithography using a polydimethylsiloxane (PDMS) mold, they optimized the preparation process, which greatly reduced the production cost. In addition, the sensitivity of the MRR detector was improved by more than 10 folds. Furthermore, they introduced a protective layer to shield the detector from potential contaminants by encapsulating it in a 5 μm thick UV-curable PDMS thin film. The encapsulated MRR detector was exposed to physiological contaminants to evaluate its stability. As shown in Fig. 5(b), the optical resonance of the unprotected MRR decreased rapidly after being immersed in blood for 15 mins. In contrast, the encapsulated MRR showed no obvious change in detection sensitivity over 2 h. These improvements make MRR more suitable for long-term PAI.

After investigating the performance of the MRR detector, Li et al. [69] first applied it to the PAI of the cortical vasculature in a living mouse brain with the skull removed. Figure 5(c) shows a photograph of a typical chronic cranial window fabricated with MRR. As shown in Fig. 5(d), combined with the MRR detector, PAM is suitable for visualizing the cortical vasculature with a high axial resolution of 3.57 μm. In addition, they performed longitudinal PAM imaging of cortical vasculature using a cranial window fabricated with a newly designed MRR detector. To evaluate the stability of the MRR detector in vivo, Figs. 5(e-h) present four PA images of the typical mouse brain at 0, 7, 14, and 28 days post-implantation, which demonstrates that the Q-factor of the cranial window reduces marginally after 28 days. An obvious neovascularization process can be observed in the regions marked by yellow dashed boxes. These results confirm that the ultrasound-sensing chronic cranial window is suitable for long-term PAI.

### PDMS film-based chronic cranial window for PAM

**Fig. 6:**
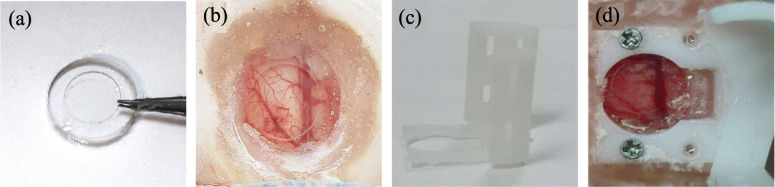
**a** Photograph of the PDMS-based cranial window implant [62]. **b** A photograph of the cranial window for PA imaging in an anesthetized rat. **c** A photograph of the 3D-printed resin frame. **d** A photograph of the cranial window for PA imaging in a freely moving rat

A previous investigation demonstrated that optically transparent PDMS film can be used as the cranial window for long-term optical imaging [40]. However, such a PDMS film is thick, which greatly attenuates the ultrasonic waves and prevents its direct use in PAI. To overcome the acoustic attenuation caused by the impedance mismatch between the brain tissue and the thick PDMS film, Wang et al. [62] introduced a newly designed PDMS-based cranial window implant allowing a large imaging area with a diameter of 5 mm. As shown in Fig. 6(a), the cranial window implant was fabricated using a PDMS layer and a covering glass. They compared the cranial window imaging quality using different thicknesses, and demonstrated that the signal-noise ratio (SNR) can be decreased by 30% when the thickness of the PDMS film exceeds 400 μm. The chosen thickness of the PDMS layer (approximately 50 μm) was sufficiently thin for an effective transmission of the ultrasonic signal. Furthermore, considering the elastic deformation capability of the PDMS film, it was bonded with a glass ring via air plasma cleaning to avoid potential deformation and wrinkles.

**Fig. 7:**
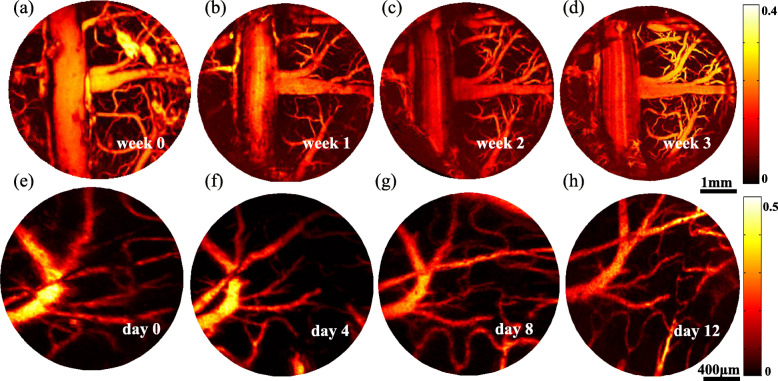
Long-term in vivo PAI in both anesthetized and freely moving rats [62]. **a**-**d** Four typical PA images captured at 0, 1, 2, 3 weeks post-implantation in anesthetized rats. **e**-**h** Four typical PA images captured at 0, 4, 8, and 12 days post-implantation in freely moving rats

To evaluate the stability of longitudinal PAI, a long-term window was fabricated in rats (Fig. 6(b)). Figure 7(a-d) shows four typical PA images of the cerebral vasculature of a rat brain captured at 0, 1, 2, and 3 weeks post-implantation, which demonstrates that the cortical vasculature can be stably detected up to 3 weeks post-implantation. In addition, quantitative analyses of both total vascular number and signal background ratio also revealed the stability of this cranial window.

By integrating with a 3D-printed resin frame [Fig. 6(c)], the cranial window was successfully applied to brain imaging of freely moving rats [Fig. 6(d)]. Figure 7(e-h) show four typical PA images of the cerebral vasculature of a moving rat obtained at 0, 4, 8, and 12 days post-implantation. The results indicate that the same brain region can be precisely monitored over a period of 2 weeks.

The imaging results in both anesthetized and freely moving rats revealed that the chronic cranial window provides an available method for long-term observation of cortical vasculature, offering promising opportunities for fundamental brain investigations. With this window model, various cerebral disorders, including stroke, epilepsy, brain tumors, and Parkinson’s disease can be further investigated.

## Conclusion and discussion

This paper reviews the reported cranial windows for long-term in vivo imaging of PAM. The scattering and attenuation of both light and ultrasound caused by the turbid skull usually result in inaccurate information on neuron activities or cerebral hemodynamics by severely reducing the resolution and SNR. Various chronic cranial windows have been fabricated to overcome the adverse effects of light caused by the skull, enabling researchers to perform longitudinal optical imaging [39, 40, 43]. However, to the authors’ knowledge, although these optical cranial windows will benefit PAM, which is based on the principle of all-optical detection, it does not compensate for the ultrasonic attenuation caused by the acoustic impedance mismatch at the window interface, preventing its broad application in PAI. Fortunately, inspired by these window techniques, several innovative chronic cranial windows have been proposed for long-term PAI [42, 61, 62]. Table 1 shows a detailed comparison of technical parameters, including the cranial window technique, lateral resolution, axial resolution, imaging depth, and stability. In this section, the current limitations and required improvements of the PA cranial window are discussed for further development.

**Table 1:** The systemic parameters of chronic cranial window for PAI

Cranial window technique	Group	Later resolution	Axial resolution	Imaging depth	Stability
**Optical cleaning of window**	**Yang et al.** [61]	**45 μm**	**15 μm**	**Approximately 3 mm**	**> 1 h**
**Ultrasound-sensing window**	**Li et al.** [42]	**Approximately 2.1 μm**	**Approximately 3.6** **μm**	**–**	**28 days**
**PDMS film based window**	**Wang et al.** [62]	**Approximately 10 μm**	**Approximately 90 μm**	**Approximately 0.8 mm**	**21 days**

Skull optical clearing, as an emerging cranial window technique, is derived from TOC, enabling scientists to image the cerebral hemodynamics with an intact skull using PAM [61]. However, further improvements are required to achieve better long-term imaging results in living animals. For example, although this technique can improve the ultrasonic transmission of the skull, to date it is only available for mice, which have a thin skull. Owing to the technique’s non-ignorable ultrasound attenuation in thick skulls, further investigations should be performed in rats and other thick-skulled large animals. Moreover, the safety of the skull window technique should be taken into account if the SOCS produces inflammation of the cortex and damages the cerebral vasculature, even neurons. In addition, the skull window requires repetitive treatment to achieve long-term brain imaging. Further studies should be conducted to reduce the treatment time to avoid potential damage to the animals.

With regard to the ultrasonic-sensing chronic cranial window, an optically transparent MRR ultrasonic detector with a detection bandwidth of over 280 MHz is used for long-term in vivo PAI in mice brains [42]. The axial resolution of this detector is up to 2.12 μm, and enables the researchers to obtain three-dimensional high-resolution images of the cerebral vasculature. However, investigation of this window technique is in still in the initial stages. The technique still faces the challenge of acoustic impedance mismatch between the window and brain tissue owing to the introduction of cover glass, which limits its application in PAM using ultrasonic transducers. Further studies should focus on realizing a broader application of this chronic cranial window. For instance, the MRR detector is ultrasensitive to disturbances from the external environment, and the stability of the MRR detector should be considered to obtain a high SNR image. Moreover, a larger field of view is required to enable the imaging of a wider range of cortical vessels. In addition, the fabrication of MRR detectors usually requires complex nanoimprint lithography processes.

The PDMS film-based chronic cranial window has excellent repeatability owing to the simple fabrication process of the implant [62]. In addition, by using a thin PDMS layer as the implant, the acoustic impedance mismatch between the brain tissue and implant can also be resolved, which enables its application to most existing PA microscopes. Although this cranial window has been proven to be suitable for PAI in both anesthetized and freely moving rats, further improvements should be considered. For instance, the window should be maintained for a longer time to enable long-term PAI.

In summary, the chronic cranial window technique, as an effective auxiliary tool, provides a better understanding of brain activity. Its continuous development and optimization will play an increasingly important role in brain investigations.

## Data Availability

NA

## Abbreviations

LSCI: Laser speckle contrast imaging
tMCAO: Transient middle cerebral artery occlusion
PAI: Photoacoustic imaging
PA: Photoacoustic
TOC: Tissue optical clearing
OR-PAM: Optical-resolution photoacoustic microscopy
PAM: Photoacoustic microscopy
SOCS: Skull optical cleaning solution
MRR: Micro-ring resonator
PDMS: Polydimethylsiloxane
SNR: Signal-noise ratio
USAF: United States Air Force
